# Genetic correlates of autoreactivity and autoreactive potential in human Ig heavy chains

**DOI:** 10.1186/1745-7580-5-1

**Published:** 2009-02-27

**Authors:** Joseph M Volpe, Thomas B Kepler

**Affiliations:** 1 Center for Computational Immunology, Duke University, Durham, NC, USA; 2 Departments of Biostatistics & Bioinformatics and Immunology, Duke University Medical Center, Durham, NC, USA; 3 Computational Biology and Bioinformatics Graduate Program, Institute for Genome Sciences and Policy, Duke University, Durham, NC, USA

## Abstract

**Background:**

Immature bone marrow B cells are known to have longer CDR3 than mature peripheral B cells, and this genetic characteristic has been shown to correlate with autoreactivity in these early cells. B-cell Central tolerance eliminates these cells, but it is known that autoreactive B cells nevertheless appear commonly in healthy human blood. We examined over 7,300 Ig genes from Genbank, including those annotated by their discoverers as associated with autoreactivity, to determine the genetic correlates of autoreactivity in mature B cells.

**Results:**

We find differential biases in gene segment usage and higher mutation frequency in autoreactivity-associated Ig genes, but the CDR3 lengths do not differ between autoreactive and non-autoreactive Ig genes. The most striking genetic signature of autoreactivity is an increase in the proportion of N-nucleotides relative to germline-encoded nucleotides in CDR3 from autoreactive genes.

**Conclusion:**

We hypothesize that peripheral autoreactivity results primarily from somatic mutation, and that the genetic correlates of autoreactivity in mature B-cells are not the same as those for autoreactivity in immature B cells. What is seen in mature autoreactive B cells are the correlates of *autoreactive potential*, not of autoreactivity per se. The autoreactive potential is higher for V(D)J rearrangements encoded to a large extent by N-nucleotides rather than by the gene segments that, we posit, have been selected in germline evolution for their suppression of autoreactive potential.

## Background

Self-reactive immunoglobulins (Ig) are generated at high frequency during normal B-cell ontogeny in the bone marrow. Functional Ig genes result from the combinatorial joining of gene segments from two (light chain) or three (heavy chain) classes; V (variable), D (diversity), and J (joining) [1,2]. This process, known as V(D)J recombination, generates tremendous receptor diversity through the pairing of various gene segments, the selection of the recombination sites at which the segments are joined, and the addition of non-templated nucleotides (n-nucleotides) between adjoining gene segments. These processes are stochastic and can produce 10^14 ^or so different protein specificities to be generated from the fewer than 100 gene segments at the immunoglobulin heavy chain locus [3]. In response to antigenic stimuli, mature B-cells undergo further diversification through somatic hypermutation (SHM), whereby mutations are introduced into the rearranged immunoglobulin gene at a rate approximately 10^6 ^times higher than the normal background rate [4]. The adaptive immune system generates diversity and adapts its antigen receptors via stochastic somatic processes as microbes themselves diversify and adapt through Darwinian evolution. With such randomness in the formation of antigen receptor genes, it is inevitable that autoreactivity will arise. Indeed, 55–75% of early bone-marrow B-cells express polyreactive and self-reactive surface Ig [5]. Three primary B-cell-specific mechanisms for avoiding Ig-mediated autoimmunity have been identified: selective deletion, anergy and receptor editing [6-10].

Experiments using site-directed mutagenesis and CDR3 replacement have shown that the heavy chain CDR3 provides the primary structural correlates of polyreactivity [11,12]. Shiokawa et al [13] found that CDR3 length differs between neonates and adults and provided evidence for somatic selection on CDR3 length during B-cell development. Aguilera et al [14] examined 8 autoreactive monoclonal antibodies, and found no clear relationship between polyreactivity and heavy chain CDR3 length. Wardemann et al [5] pooled immature and bone marrow "new emigrant" B-cells to show that cells reactive to HEp-2 cell exctract are biased toward longer CDR3 compared to those that are not so reactive.

Autoantibodies are commonly present in healthy human serum [15]; in murine models, positive selection for some autoreactive antibodies has been observed [16]. It is possible that these autoantibodies result largely from "leakiness" of negative selection in the bone marrow, in which case one expects autoantibodies in the periphery to bear the same genetic signatures as autoantibodies in immature B cells. But they may also arise in the periphery via somatic mutation after central tolerance has acted. In this case, we might expect to find a genetic signature of autoreactivity, which may differ from that common to pre-selection autoreactive cells in the bone marrow.

To determine the genetic signature of peripheral autoreactivity, we performed a comprehensive and comparative study of over 7,300 Ig collected from Genbank. We assembled four sets of human heavy chain genes for comparison: a set of productive Ig genes for which evidence of autoreactivity does not exist (P); a heterogeneous set of genes annotated by their discoverers as associated with autoreactivity, but exlcuding rheumatoid arthritis (A); a set genes annotated as associated with rheumatoid arthritis (RA); and a set of genes (NP) that appear to have been rearranged out-of-frame and thus nonproductively.

We performed a detailed analysis of each gene set in terms of gene segment composition, CDR3 length, n-nucleotide addition, and mutation frequency, and employed statistical methods to detect biases that may exist among these sets. We find differential biases in gene segment usage and n-nucleotide tract length, but not in CDR3 length between autoreactive and non-autoreactive productive genes. We do, however, find a striking increase in the proportion of n-nucleotides in CDR3 from autoreactive genes. This fact suggests to us that germline gene segments have evolved under selective pressure, not only to avoid autoreactivity, but to avoid acquiring autoroactivity through somatic mutation. Consequently, autoreactivity in the periphery arises more frequently by somatic mutation in those genes in which the CDR3s are encoded more extensively by n-nucleotides rather than germline gene segments.

## Results

### Gene Segment Usage

**Figure 1:**
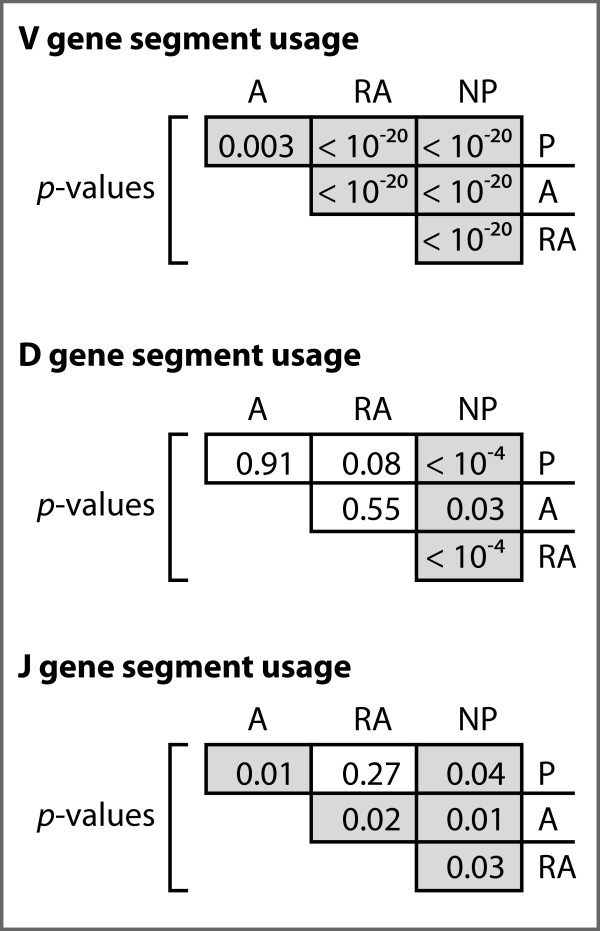
**Analysis of gene segment frequencies among the four datasets**. P-values for chi-square analyses between all combinations of sequence sets. P-values lower than 0.05 are highlighted in gray.

**Figure 2:**
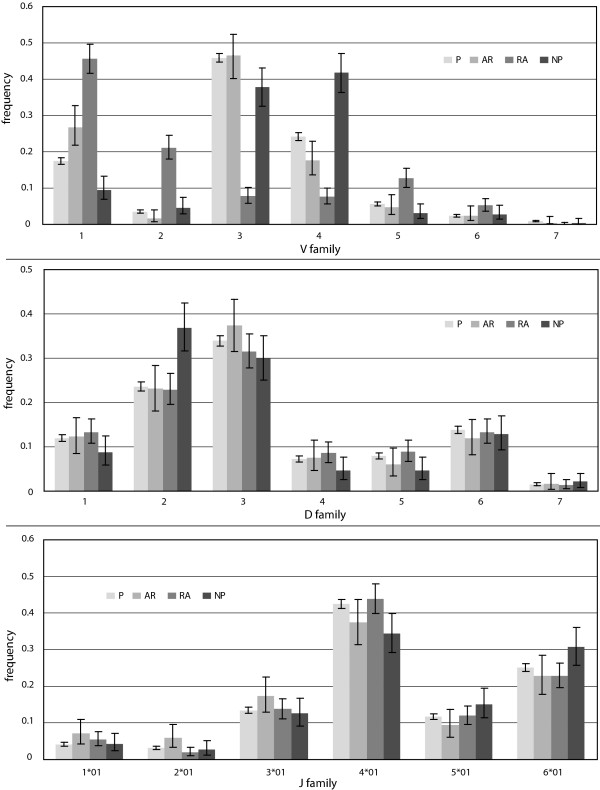
**Gene segment usage frequencies**. Observed frequencies of gene segment usage in the P, A, RA, and NP gene sets. Error bars represent 95% confidence intervals.

For the J segments, both the A and NP gene sets showed significant differences compared to every other gene set, as did the RA and P gene sets, except when compared to each other (Figures 1, 2). All possible pairs among the four gene sets showed statistically significant J-gene segment usage differences except for the RA vs P gene comparison.

The V and D segments outnumber J segments by factors of eight and four, respectively, and are divided into seven families each based on sequence homology. Looking at the frequency of V segment usage by family, we observe statistically significant differences among all gene sets (Figures 1, 2). For both the A and RA genes, the most notable observation is the increase of 50% and 130%, respectively, of segments from family VH1 relative to normals, but a 26% and 67% decrease, respectively, in the relative use of segments from family VH4 (Figure 2). The RA genes also showed dramatic enhancements in the usage of segments from family VH2 and VH5 by four- and two-fold, respectively, relative to the P genes (Figure 2). For the NP genes, we observed the opposite trend in family VH4, with 67%, 115%, and 34% relative increases of segment usage relative to the P, A, and RA genes, respectively.

Among the productive gene sets, we did not observe statistically significant differences in D segment usage by family (Figures 1, 2). The NP genes, however, did differ significantly from each of the three productive gene sets. We also observed significant enhancements of 33% and 136% in the number of apparent inverted D-segments used in the A and RA genes, respectively, relative to the P genes (*p *= 0.02).

### Somatic Mutations

**Figure 3:**
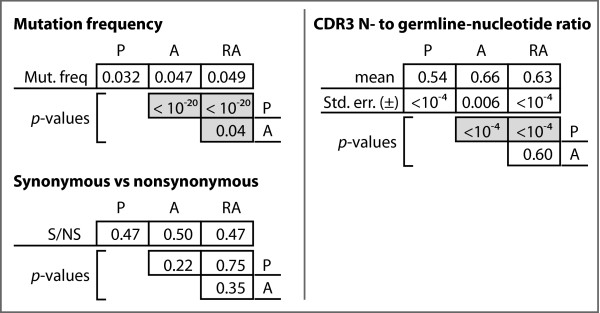
**Analysis of somatic mutations and N-nucleotides among the functional datasets**. P-values for chi-square analyses between the functional Ig datasets with respect to mutation frequency, mutation types, and ratio of N-nucleotides to germline-encoded nucleotides.

We observed significantly higher mutation frequencies of 4.7% and 4.9% for the A and RA genes, respectively, compared to the P genes (*p *< 10^-10^). These values represent a 46% and 47% relative increase over the observed mutation frequency for the P genes of 3.2%. Despite higher mutation frequencies, we did not observe a significant difference in the ratio of non-synonymous to synonymous mutations in the three gene sets (Figure 3). Mutation frequencies were calculated from observed mutations within the V segment only of each Ig gene.

### CDR3 length

**Figure 4:**
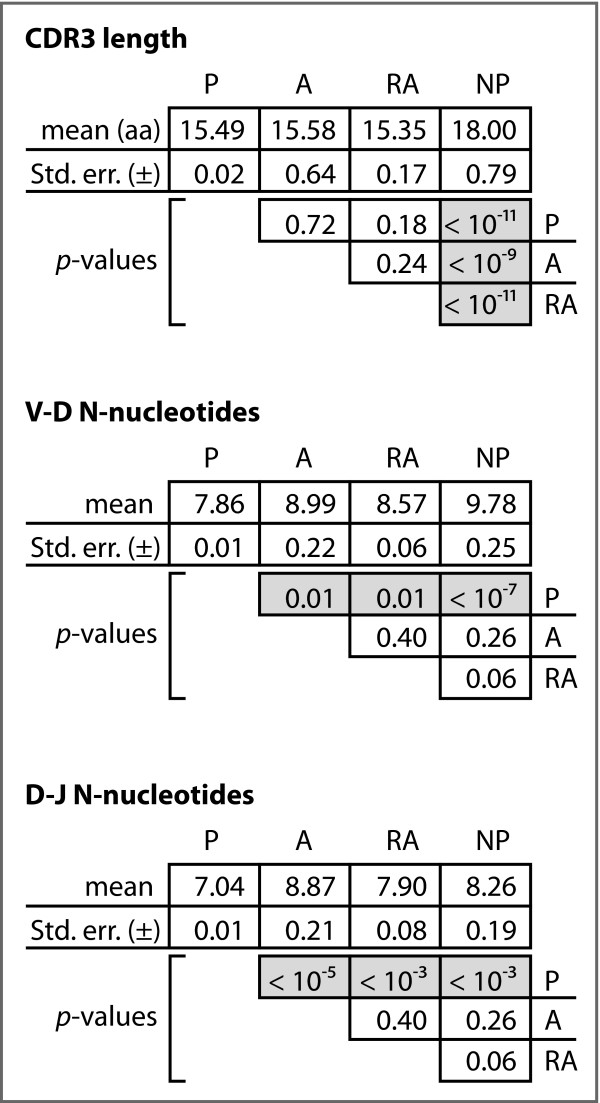
**Analysis of CDR3**. Mean numbers of N-nucleotides in the two junctions, and mean CDR3 length. p-values are based on chi-square analyses.

**Figure 5:**
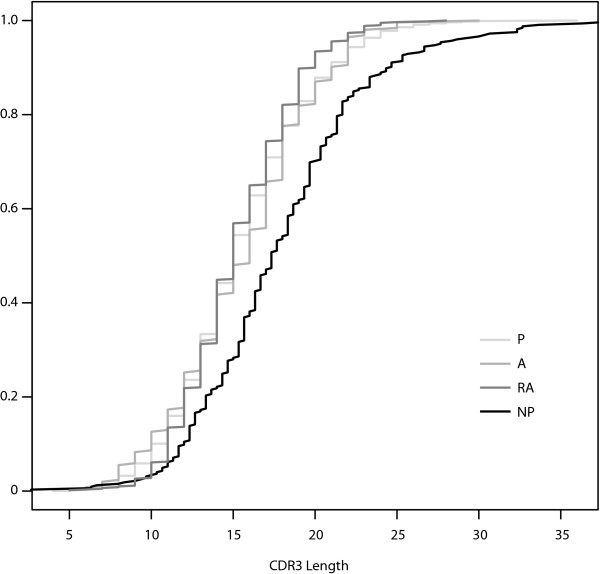
**Cumulative Distribution Functions for CDR3 Length**. The empirical cumulative distribution functions for CDR3 length for the four datasets plotted together. The functional gene sets, P, A, and RA, all have similar cumulative distribution functions for CDR3 length.

We observed mean CDR3 lengths of 15.49 aa, 15.58 aa, and 15.35 aa for the P, A, and RA genes, respectively (Figure 4). The differences among these means are not statistically significant. We did, however, observe a statistically significantly increased CDR3 length in the NP genes compared to each of the productive gene sets. Plotting the cumulative distribution functions for the CDR3 data for each gene set shows that the CDR3 lengths of the P, A, and RA genes are distributed similarly, while the distribution of lengths for NP gene CDR3 lengths differs (Figure 5).

### N-nucleotide Frequency

We observed significant differences for the mean N-nucleotide tract lengths in both the V-D and D-J junctions among the four gene sets (Figure 4). In particular, the A, RA, and NP sequences differed significantly from the P sequences, with relative increases of 14%, 9%, and 24%, respectively, in the V-D junction and relative increases of 26%, 21%, and 17%, respectively, in the D-J junction. In neither junction did we observe differences among the A, RA, and NP sequences sets.

We further computed the ratio of N-nucleotides to germline-encoded nucleotides in each of the three productive gene sets. Figure 4 shows the mean ratios and indicates that there is a statistically significant difference in these mean ratios between the A and RA genes relative to the P genes, but that no such difference exists between the A and RA genes.

## Discussion

In spite of selection against B cells bearing autoreactive antigen receptors during ontogeny in the bone marrow, substantial frequencies of autoreactive B cells in the periphery are observed. There are two hypotheses, not mutually exclusive, to account for this fact. One such hypothesis holds that such selection is intrinsically leaky, and that autoreactivity in the periphery is due largely to original autoreactivity that has slipped through the selective filter. The other hypothesis does not depend on leakiness of central B cell tolerance, but instead relies on the generation of autoreactivity by somatic mutations incorporated after primary B cell development.

These two hypotheses make different predictions regarding the relationships one will find between the genetic signatures of autoreactive Ig in the bone marrow and those in the periphery. If peripheral autoreactivity is due primarily to the escape of original autoreactivity from central tolerance, we expect to find that autoreactive peripheral B cells will be similar to pre-selection and autoreactive B cells in the bone marrow. The main distinguishing feature of such cells is a long CDR3. We find no statistically significant difference between CDR3 lengths in autoreactive and non-autoreactive Ig, though CDR3 is much longer in NP sequences than in any other class, confirming the observation by others that selection during primary ontogeny favors shorter CDR3.

No such correspondence is necessary under the second hypothesis. What one does expect to see under the second hypothesis are differences in somatic mutation, and we do indeed find that the mutation frequency is about 50% higher in autoreactivity-associated genes than in non-autoreactive genes. This result is consistent with many previous studies of the role of somatic mutation in autoimmunity [17-22]. Beyond the simple observation of differential mutation frequencies, however, we do see additional genetic biases that distinguish the autoreactives from the normals.

We observe, for example, differential usage in both VH and JH gene segments, with an increase in VH1 family usage in genes from both the general autoimmune (A) and rheumatoid arthritis (RA) datasets, as well as a corresponding reduction in family VH4 usage. Family VH4 is typically second-highest in usage frequency and has been reported to provide some of the most frequently used segments in the adult Ig repertoire, particularly V4-34 [23-26].

If peripheral autoreactivity were due largely to the leakiness of central tolerance, we would expect gene segment usage in autoantibodies to trend toward the pre-selection repertoire represented by the non-productively rearranged genes. This is not what we find. In fact, the VH segment use in the A and RA genes is not intermediate between the NP and normal genes, it is less like that of the NP genes than the normals are. VH1, for example, has a higher frequency in the normal genes than in the NP genes, and higher frequency still in the A and AR genes, while VH4 is lower in normals than in NP and lower still in A and AR.

The selective enhancement of usage for VH1 and VH5 segments in the A and RA genes may be due partially to the presence of charged residues in the framework region of these gene segments. Studies of autoimmune anti-erythrocyte human protein antibodies and non-autoimmune anti-staphylococcal antibodies show that framework region 1 may play a critical role in effective binding to these antigens [27,28]. The VH1 and VH5 family genes contain a lysine-lysine amino acid motif in framework 1, which would produce a positively charged region in the folded protein. No other VH segment contains a contiguous pair of positively charged residues. We did also observe an enhancement of VH5 segment usage in the RA genes. Thus, it may be possible that part of the framework region 1 of the VH1 family genes promotes binding to some self-antigens.

We did observe, however, significant differences in the proportions of N-nucleotides among CDR3 nucleotides between either autoreactive set and the normals. This result is consistent with those from studies showing that TdT-deficient mice crossed with autoimmune-prone mice, which make B- and T-cells with nearly no N-nucleotides, have lower incidence of autoimmune disease and longer life spans than non-TdT deficient autoimmune-prone controls [29,30].

These analyses suggest to us that the premutation genetic signatures observed in the autoreactive sets are indicative of *autoreactive potential*, and mark those genes that are more likely to give rise to autoreactivity under somatic mutation. B-cell central tolerance can only counter-select V(D)J rearrangements that are already autoreactive, not those with a tendency to become autoreactive. Germline evolution, however, can and does select gene segments for their potential repertoire under somatic mutation [31,32].

The idea is that germline VH, DH, and JH gene segments have been shaped by evolution to avoid becoming autoreactive too easily under somatic hypermutation. When much of CDR3 is encoded by N-nucleotides rather than by these time-tested gene segments, autoreactivity may be much closer in genotype space than it would otherwise be.

## Conclusion

We have examined the genetic correlates of B cell autoreactivity by compiling a large set of Ig genes associated with autoimmunity and comparing these genes statistically to normal and non-productive Ig gene sequences. We do not find a difference in CDR3 length between the autoimmunity-associated genes and the normals, as one might expect. There are, however, marked differences in mutation frequency between these sets, differences in gene-segment usage frequency, and, most strikingly, an increased use of N-nucleotides in the autoimmune genes. The hypothesis that ties these results together is that the premutation genetic signature of peripheral B-cell autoreactivity is not related to autoreactivity per se (because autoreactivity is largely eliminated by counter-selection in the bone marrow) but to autoreactive potential, the proclivity to become autoreactive under somatic hypermutation.

## Methods

### DNA sequences

We compiled two sets of human immunoglobulin heavy chain gene sequences intended to be representative of the the naturally-occurring adult human repertoires. We excluded clonally-related genes and genes of perinatal origin. To do so, we submitted the search "human [orgn] heavy [titl] immunoglobulin [titl]" to the Genbank nucleotide database which returned 16,870 results. We further identified 1,167 autoreactive Ig rearrangements in Genbank using keyword searches with terms such as "autoreactive", "immunoglobulin", "autoantibody", and "heavy". We also identified a third set of 608 gene sequences from a study conducted on the synovial B-cells of rheumatoid arthritis (RA) patients [33]. We downloaded each set of DNA sequences, preprocessed and filtered them as described below, and analyzed them for gene segment usage, point mutations, n-nucleotide addition, and recombination junctional diversity. Note that the last nucleotide (or more) consistent with the relevant gene segment may be a "cryptic" N-nucleotide. We consistently identify such nucleotide as germline-encoded in spite of this uncertainty. The larger set would be designated as productive, non-autoreactive genes (P), while the smaller set would be designated as productive, autoreactive genes (A). The automated analysis was performed using our in-house software SoDA [34].

### Classification by Productivity

Each dataset was divided into two groups on the basis of their inferred original, pre-somatic mutation productivity. We classified those sequences that had no stop codons and both invariant V cysteines and the invariant J tryptophan in-frame and intact as productive. Those that appeared to have been originally rearranged out of frame by virtue of the VH segment being out of frame with the JH segment, excluding indels, were classified as non-productive (NP). All others were classified as indeterminate and were omitted from further consideration.

### Filtering

We then filtered the SoDA results in each of the four sets to remove clonal duplicates, which we defined to be those sequences that were inferred to use the same V, D, and J gene segments, had the same inferred CDR3 length, and have similar Genbank accession numbers. Sequences containing these matches and differing only by point mutations were still considered clonal duplicates. Where groups of clonally related genes were identified, a single representative was chosen at random and the others were omitted. We then grouped the sequences in each set by study of origin and removed any large sets of sequences that came from the same study.

The P sequences were further filtered to remove those sequences that, by their own Genbank annotations, indicated origin from neonates or cordblood. The P sequences were also filtered to remove any sequences that may be autoreactive due to the inclusion of at least one of the following words in the Genbank record: "self-reactive", "anti-self", "lupus", "rheumatoid", "sjogren", "diabetes", "sclerosis", "wegener", "crohn", "addison", "scleroderma", "grave", "psoriasis", "celiac", "vasculitis", "colitis", and "thyroiditis".

Each autoreactive sequence was then manually verified to ensure that its Genbank record indicated autoreactivity. The A dataset was also filtered to remove those genes that were specifically anti-DNA, since these genes would bias the CDR charge measurements. The final set of P genes contained 6490 DNA sequences, the final set of A genes contained 254 sequences, the final set of RA sequences from the rheumatoid arthritis study contained 608 sequences, and the final set of NP sequences contained 325 genes.

## Competing interests

The authors declare that they have no competing interests.

## Authors' contributions

JMV gathered and analyzed the data, and drafted the manuscript. TBK determined, and where necessary, developed the appropriate statistical strategies needed for the presented analyses. Both authors contributed to the final writing of the manuscript.
